# Operative hysteroscopy intravascular absorption (OHIA) syndrome presenting with severe hypoxemia and abnormal carboxyhemoglobin elevation

**DOI:** 10.12669/pjms.42.9.13717

**Published:** 2026-09

**Authors:** Dongmiao Cai, Haizhen Wang, Bin Yang, Zhihong Xu

**Affiliations:** 1 Dongmiao Cai, Department of Anesthesiology, The First Affiliated Hospital of Xiamen University, School of Medicine, Xiamen University, Xiamen, China, 361000; 2 Haizhen Wang, Department of Anesthesiology, The First Affiliated Hospital of Xiamen University, School of Medicine, Xiamen University, Xiamen, China, 361000; 3 Bin Yang, Department of Anesthesiology, The First Affiliated Hospital of Xiamen University, School of Medicine, Xiamen University, Xiamen, China, 361000; 4 Zhihong Xu, Department of Anesthesiology, The First Affiliated Hospital of Xiamen University, School of Medicine, Xiamen University, Xiamen, China, 361000

**Keywords:** Carboxyhemoglobin, OHIA, Hypoxemia

## Abstract

A 40 years old woman undergoing elective hysteroscopic myomectomy under general anesthesia developed progressive hypoxemia and severe metabolic acidosis. The primary etiology was Operative Hysteroscopy Intravascular Absorption (OHIA) syndrome, resulting from massive normal saline irrigation (approximately 18 L used) leading to dilutional hyperchloremic metabolic acidosis and pulmonary edema. Intraoperatively, a sudden drop in end-tidal carbon dioxide (EtCO_2_) and an incidental elevation in carboxyhemoglobin (COHb) of 15.7% were noted. This COHb elevation was suspected to be due to spectrophotometric interference by dissolved hydrogen gas generated during bipolar electrosurgery, which might have indirectly suggested a concurrent, transient venous gas embolism. However, OHIA remained the principal driver of hypoxemia and clinical deterioration. Management included fluid restriction, diuretics, steroids, and supportive ventilation. The patient recovered uneventfully. This case highlights the dominant role of OHIA in prolonged high-pressure hysteroscopy, with artifactual COHb elevation serving as a potential (though non-definitive) ancillary clue to intravascular gas entry.

## INTRODUCTION

Hysteroscopic surgery is common, with major complications including OHIA syndrome, uterine perforation, hemorrhage, and air/gas embolism. Incidence (0.22%-3.7%) correlates with operative duration, extent of surgical resection, distension pressure, irrigation fluid type, surgeon skill, and patient comorbidities.^1^ OHIA syndrome, first described in 1993, shares similarities with transurethral resection of the prostate (TURP) syndrome, presenting with fluid overload, pulmonary edema, heart failure, dilutional anemia, and hyper/hyponatremia.^2^ Uterine perforation and hemorrhage are typically readily recognized and addressed. Transesophageal echocardiography studies classifying gas embolism (Grade 0-IV) found venous emboli in 98% of patients undergoing mono/bipolar hysteroscopy, though overt complications were rare.^3^ A literature review (January 2000-May 2020) identified only five reported cases of air embolism during hysteroscopic myomectomy using bipolar resection with saline distension. Outcomes varied: one unsuccessful resuscitation attempt via central venous catheter, one transient left lower limb sensory deficit with small left parietal cortical infarction, one pulmonary embolism, and two without significant sequelae.^4^ This report details a case of hypoxemia with abnormally elevated COHb and hyperchloremic metabolic acidosis during hysteroscopic myomectomy, and discusses the potential link between COHb artifact and gas embolism.

## CASE PRESENTATION

### Preoperative Assessment:

A 40 years old female (height 158 cm, weight 61 kg, BMI 24.43 kg/m²) was admitted on July 11, 2025, for “irregular vaginal bleeding for four months.” Admission ultrasound revealed an enlarged uterus with fibroids (largest 1.2x1.0 cm on posterior wall), a 6.0x4.4 cm submucosal fibroid, and a right ovarian cyst (4.0x3.2 cm). Preoperative investigations were unremarkable. Medical history included seven years of hypertension (controlled with Losartan 50mg daily), iron deficiency anemia (treated with Trifercaps® Ferrous Fumarate, Vitamin C & Folic Acid), two prior cesarean sections (2009, 2014), and hysteroscopic polypectomy (April 2025). Admission diagnosis was submucosal fibroid, scheduled for elective hysteroscopic myomectomy under general anesthesia. Pre-anesthetic assessment classified her as American Society of Anesthesiologists (ASA) physical status II with no contraindications.

### Intraoperative Course:

Baseline vitals upon OR arrival: BP 170/89 mmHg, HR 65 bpm, RR 16 bpm, SpO_2_ 100%. Anesthesia was induced with midazolam 2mg, propofol 3mg/kg, lidocaine 50mg, sufentanil 20µg, and cisatracurium 8mg. A size 3.0 Laryngeal Mask Airway(LMA) was inserted after loss of eyelash reflex. Anesthesia was maintained with propofol Target-Controlled Infusion(TCI) (3µg/ml plasma), remifentanil TCI (2ng/ml effect-site), and 1% sevoflurane. Ventilation maintained EtCO_2_ between 35-45 mmHg. Post-induction vitals: BP 87/44 mmHg, HR 77 bpm, RR 12 bpm, SpO_2_ 100%, EtCO_2_ 43 mmHg, Peak Airway Pressure (PAP) 5-6 cmH_2_O. Surgery commenced with distension pressure ~120 mmHg, flow rate 300 ml/min (normal saline irrigation). A large submucosal fibroid was visualized, requiring extensive resection.

### At 20 minutes:

EtCO_2_ abruptly decreased from 38 mmHg to 27 mmHg within two minutes, HR increased from 55 to 83 bpm, and BP dropped from 97/51 mmHg to 83/43 mmHg. Ephedrine 6mg IV was administered, improving BP transiently. EtCO_2_ remained low (27-28 mmHg), SpO_2_ decreased to 93-94%. Auscultation revealed scattered bilateral wheezes; brief runs of ventricular bigeminy occurred. The surgeon confirmed that there was no uterine perforation and initially suspected OHIA syndrome. FiO_2_ was increased to 100%, manual lung recruitment maneuvers performed, and dexamethasone 10mg, cisatracurium 4mg, and furosemide 10mg IV were given; sevoflurane was discontinued. SpO_2_ improved to 98-99%. BP fluctuated between 88/56 and 108/54 mmHg.

### At 50 minutes:

Progressive SpO_2_ decline to 90% and EtCO_2_ decline to 15 mmHg occurred. The LMA was removed, and the patient was intubated with a 6.5mm Endotracheal Tube(ETT). Manual ventilation yielded minimal CO_2_ waveform (EtCO_2_ ~11 mmHg) and high PAP (24-26 cmH_2_O). Auscultation revealed diffuse bilateral rales; SpO_2_ plummeted to 66%. Suspecting pulmonary air embolism (PAE), surgery was halted, and the patient was positioned in left lateral Trendelenburg. Ephedrine 12mg and epinephrine 20µg IV boluses, plus methylprednisolone 40mg IV, were administered. Vital signs stabilized: BP 105/64 - 138/74 mmHg, HR 80-89 bpm, SpO_2_ 99-100%, EtCO_2_ 30-31 mmHg. Right internal jugular venous and radial arterial lines were placed for monitoring and Arterial Blood Gas(ABG) sampling.

### Intraoperative ABG Results:

**Table:** 

Parameter	10:06 AM	10:19 AM	10:40 AM
PH	7.101	7.148	7.276
PCO₂ (mmHg)	56.50	54.80	49.60
PaO_2_ (mmHg)	281 (FiO₂=1.0)	264 (FiO₂=1.0)	239 (FiO₂=1.0)
P(A-a)O_2_ (mmHg)	364.30	383.40	413.50
K^+^(mmol/L)	2.80	3.10	3.10
Ca^2+^(mmol/L)	0.92	0.91	0.86
Na^+^(mmol/L)	146.00	146.00	148.00
Cl^-^(mmol/L)	146.00	146.00	148.00
BE(mmol/L)	-12.10	-9.90	-3.80
HCO₃(mmol/L)	15.10	16.70	21.30
Hct(%)	30.00	30.00	30.30
Hb(g/dL)	9.80	9.80	9.90
HbO₂(%)	83.30	83.70	86.70
COHb(%)	15.70	14.70	12.40
MetHb(%)	0.90	1.30	1.00
HHb(%)	0.10	0.30	0.10
Glu(mmol/L)	6.90	6.90	7.50
Lac(mmol/L)	0.80	0.70	1.00

***Abbreviations:*** **BE:** Base Excess; **Hct:** Hematocrit; **MetHb:** Methemoglobin; **HHb:** Deoxyhemoglobin; Glu,glucose; **Lac:** Lactate.

### Postoperative Course (PACU):

Extubation occurred 30 minutes post-arrival. Vital signs were stable (BP 135/64 - 160/84 mmHg, HR 100-110 bpm, SpO_2_ 99-100%). Thirty minutes post-extubation, the patient reported chest tightness and dyspnea. Auscultation revealed diffuse bilateral rales (right > left). Bedside thoracic ultrasound showed prominent B-lines (Fig.1). Treatment included furosemide 10mg IV, hydrocortisone 100mg IV infusion, and high-flow oxygen (FiO_2_ 0.5). Urine output reached 2700 ml, resolving dyspnea and chest tightness. Transient scalp and limb paresthesia occurred. Repeat auscultation showed reduced rales. The patient was discharged to the ward.

**Fig.1:**
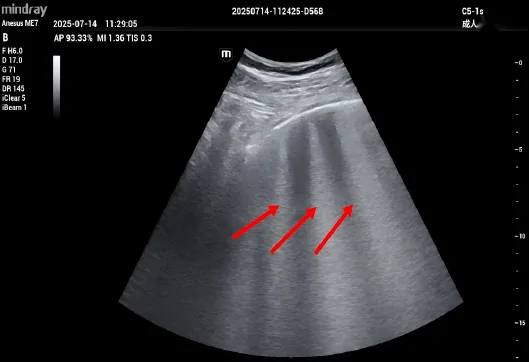
Bedside thoracic ultrasound showing prominent bilateral B-lines (arrows), consistent with interstitial pulmonary edema. These findings correlate with the patient`s clinical dyspnea and rales.

### Postoperative Day one:

Chest CT showed bilateral patchy pneumonitis and a small right pleural effusion (Fig 2).

**Fig.2:**
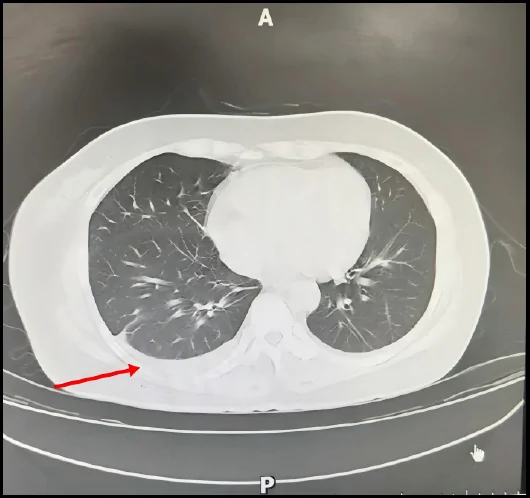
Chest CT showed bilateral patchy pneumonitis and a small right pleural effusion (arrows).

### Postoperative Day three:

The patient was discharged home.

Follow-up revealed no significant complications.

## DISCUSSION

This case illustrates a critical complication of prolonged hysteroscopic myomectomy performed under excessive distension pressure (120 mmHg). The clinical picture is best explained by a combination of severe OHIA syndrome and a presumptive, transient venous gas embolism. We discuss these entities separately below.

### OHIA Syndrome as the Primary Pathological Process:

In this case, the patient received approximately 18-19 L of normal saline irrigation, with only 200 mL of urine output and 20 mL of estimated blood loss. The arterial blood gas revealed severe hyperchloremia (Cl^-^ up to 148 mmol/L), metabolic acidosis (pH 7.101, BE -12.1), and a widened A-a gradient, all consistent with massive absorption of isotonic saline. The resulting volume overload directly caused pulmonary interstitial and alveolar edema, evidenced by diffuse bilateral rales, prominent B-lines on bedside ultrasound, and postoperative CT showing patchy opacities and pleural effusion. The absence of cardiac murmurs or ventricular dysfunction made cardiogenic edema unlikely. Therefore, OHIA syndrome was the primary cause of hypoxemia, and treatment with furosemide and fluid restriction resulted in significant clinical improvement (urine output 2700 mL and symptom resolution). A major limitation of our management was the failure to record the irrigation fluid deficit intraoperatively, which would have allowed real-time quantification of fluid absorption. We strongly advocate for automated deficit monitoring systems with alarms to prevent such severe OHIA.

### Presumptive Gas Embolism and Artifactual COHb Elevation:

In addition to OHIA, the abrupt EtCO_2_ drop (from 38 to 15 mmHg) with concomitant hypotension and tachycardia, occurring 50 minutes into surgery, is highly characteristic of venous gas embolism. Gas may have entered the circulation via opened venous sinusoids in the resected myoma bed, travelling through the uterine and iliac veins to the pulmonary circulation.^5^ While these events were self-limiting after repositioning and supportive measures, the incidental finding of elevated COHb (15.7%) on co-oximetry warrants special discussion. Bipolar electrosurgery in saline generates a complex gas mixture primarily composed of water vapor (>70%), volatile organic compounds, chlorine, and hydrogen gas (H_2_), with negligible carbon monoxide (CO).^6^-^7^ Traditional spectrophotometric blood gas analyzers may not distinguish the light-scattering effect of dissolved H_2_ at the 540 nm wavelength from true COHb, which could result in a spurious elevation of measured COHb^8^. Therefore, rather than indicating CO poisoning, this COHb spike might serve as an indirect, though non-quantifiable, sign that gas bubbles are being produced and could be entering the intravascular space. We emphasize that this is a hypothesis based on in vitro studies of electrosurgical gas production; direct measurement of H_2_ or gas chromatography was not performed. The absence of visible gas on transthoracic echocardiography does not exclude embolism, as transient microbubbles may have already dissipated by the time imaging was performed. Hence, the diagnosis of gas embolism remains presumptive, and COHb elevation should be regarded as an ancillary artifact rather than a definitive diagnostic marker.

### Differential Diagnosis and Clinical Reasoning:

*Bronchospasm:* Although transient wheezes were auscultated, the patient had no history of asthma, and the wheezes did not respond to bronchodilators but resolved with diuretics and repositioning, making bronchospasm unlikely.

### Aspiration pneumonitis:

The patient had a properly positioned LMA with no evidence of regurgitation or vomiting during anesthesia. Postoperative CT showed diffuse bilateral rather than dependent/specific lobar opacities, arguing against aspiration.

### Pulmonary thromboembolism (PTE):^9^

This is an important differential for sudden EtCO_2_ drop and hypoxemia. However, the patient had no immobilization, recent surgery (other than minor polypectomy), or hypercoagulable state. The rapid improvement with supportive measures and the absence of acute right heart strain on bedside echocardiography make PTE less probable.

### Several management issues merit reflection:

The use of distension pressure 120 mmHg likely contributed to massive fluid absorption and possible gas entry, whereas consensus recommends ≤100 mmHg or below mean arterial pressure to reduce intravasation risk. The initial EtCO_2_ drop at 20 minutes was an early warning sign that was not acted upon immediately; earlier intervention might have attenuated the severity of OHIA and pulmonary edema. The failure to track irrigation fluid deficit intraoperatively was a major limitation—a deficit exceeding 1000 ml has been associated with increased embolism risk, and automated monitoring with alarms is recommended over manual calculation. Finally, the elevated COHb reading was interpreted as a spectrophotometric artifact, likely due to dissolved H_2_ from electrosurgery, which may have supported suspicion of gas entry, while hyperchloremia indicated massive saline absorption.

## CONCLUSION

The constellation of massive fluid absorption leading to severe OHIA, with a possible transient gas embolism suggested by EtCO_2_ changes and the COHb artifact, provides the most coherent explanation for this case. The management strategy was appropriately directed towards OHIA (diuresis, steroids) while addressing the suspected embolic event (positioning, vasopressors). Effective management of such crises demands seamless communication and coordination between the surgeon, anesthesiologist, and nursing team to ensure patient safety and minimize complications.

### Ethics approval statement:

This study was approved by the Ethical Committee of The First Affiliated Hospital of Xiamen University (Approval No. 2025174; dated: September 9, 2025).

### Conflict of interest disclosure:

The authors declare no conflicts of interest.

### Funding statement:

This study was supported by the Xiamen Medical Association Clinical Research Fund (Grant No. HY202501015). The funding body had no role in the study design, data collection, analysis, interpretation, or manuscript writing.

### Acknowledgements

We thank the patient for allowing us to share her case. We acknowledge the use of DeepSeek AI (https://deepseek.com) for English language editing and polishing of this manuscript. The AI tool was used solely for grammatical and stylistic improvements; no AI tools were used for data analysis, interpretation, scientific writing, or decision-making. All clinical content, conclusions, and final approval remains the sole responsibility of the authors.

### Authors Contributions: DC and HW:

Methodology; conceptualization; data curation, formal analysis; writing – original draft, review and editing. **BY:** Analyzed and organized patient data, oversaw and approved final manuscript. **ZX:** Methodology; conceptualization; data curation, writing – editing. Dr. Zhihong Xu (corresponding author) takes full responsibility for the integrity and accuracy of the entire work.
